# The garlic compound ajoene covalently binds vimentin, disrupts the vimentin network and exerts anti-metastatic activity in cancer cells

**DOI:** 10.1186/s12885-019-5388-8

**Published:** 2019-03-20

**Authors:** Catherine H. Kaschula, Rosanna Tuveri, Ellen Ngarande, Kevin Dzobo, Christopher Barnett, Daniel A. Kusza, Lisa M. Graham, Arieh A. Katz, Mohamed Suhail Rafudeen, M. Iqbal Parker, Roger Hunter, Georgia Schäfer

**Affiliations:** 1 Department of Chemistry and Polymer Science, Stellenbosch University, Stellenbosch, 7600 South Africa; 2 Department of Biomedical Science, University of Cagliari, 09042 Monserrato, Italy; 3 Department of Integrative Biomedical Sciences and Institute of Infectious Disease and Molecular Medicine, Faculty of Health Sciences, University of Cape Town, Observatory, Cape Town, 7925 South Africa; 4 International Centre for Genetic Engineering and Biotechnology (ICGEB), UCT Medical Campus, Anzio Rd, Observatory, Cape Town, 7925 South Africa; 5 Department of Chemistry, University of Cape Town, Rondebosch, Cape Town, 7700 South Africa; 6 Department of Molecular and Cell Biology, University of Cape Town, Rondebosch, Cape Town, 7700 South Africa

**Keywords:** Antimetastatic, Cancer, Garlic, Natural product, Ajoene, EMT, Vimentin

## Abstract

**Background:**

Garlic has been used for centuries for its flavour and health promoting properties that include protection against cancer. The vinyl disulfide-sulfoxide ajoene is one of the phytochemicals found in crushed cloves, hypothesised to act by *S*-thiolating reactive cysteines in target proteins.

**Methods:**

Using our fluorescently labelled ajoene analogue called dansyl-ajoene, ajoene’s protein targets in MDA-MB-231 breast cancer cells were tagged and separated by 2D electrophoresis. A predominant band was identified by MALDI-TOF MS/MS to be vimentin. Target validation experiments were performed using pure recombinant vimentin protein. Computational modelling of vimentin bound to ajoene was performed using Schrödinger and p*K*_a_ calculations by Epik software. Cytotoxicity of ajoene in MDA-MB-231 and HeLa cells was measured by the MTT assay. The vimentin filament network was visualised in ajoene-treated and non-treated cells by immunofluorescence and vimentin protein expression was determined by immunoblot. The invasion and migration activity was measured by wound healing and transwell assays using wildtype cells and cells in which the vimentin protein had been transiently knocked down by siRNA or overexpressed.

**Results:**

The dominant protein tagged by dansyl-ajoene was identified to be the 57 kDa protein vimentin. The vimentin target was validated to reveal that ajoene and dansyl-ajoene covalently bind to recombinant vimentin via a disulfide linkage at Cys-328. Computational modelling showed Cys-328 to be exposed at the termini of the vimentin tetramer. Treatment of MDA-MB-231 or HeLa cells with a non-cytotoxic concentration of ajoene caused the vimentin filament network to condense; and to increase vimentin protein expression. Ajoene inhibited the invasion and migration of both cancer cell lines which was found to be dependent on the presence of vimentin. Vimentin overexpression caused cells to become more migratory, an effect that was completely rescued by ajoene.

**Conclusions:**

The garlic-derived phytochemical ajoene targets and covalently modifies vimentin in cancer cells by *S*-thiolating Cys-328. This interaction results in the disruption of the vimentin filament network and contributes to the anti-metastatic activity of ajoene in cancer cells.

**Electronic supplementary material:**

The online version of this article (10.1186/s12885-019-5388-8) contains supplementary material, which is available to authorized users.

## Background

**Fig. 1:**
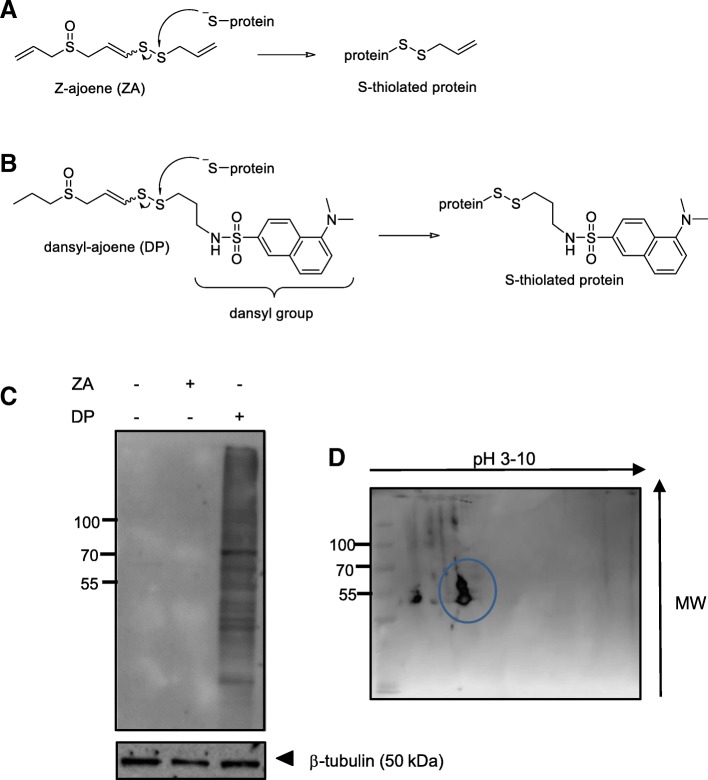
Purification and Identification of Vimentin from DP-treated MDA-MB-231 cells. Proposed disulfide exchange reaction occurring between a cysteine sulfhydryl group on a target protein with (**a**) Z-ajoene (ZA) or its analogue (**b**) dansyl-ajoene (DP). (**c**) Lysate collected from MDA-MB-231 breast cancer cells treated with 25 μM ZA or DP show many dansyl-labelled proteins by immunoblot when probed with an anti-dansyl primary antibody in the DP-treated sample only. The experiment was performed under non-reducing conditions. (**d**) Separation of the dansyl-labelled proteins in MDA-MB-231 cell lysate by 2D gel electrophoresis under non-reducing conditions. A predominant band (circled) was observed in the immunoblot which was excised from the corresponding gel and identified by MALDI-TOF MS/MS to be vimentin

Garlic (*Allium sativum*) has been used since ancient times as a food additive and for its beneficial health effects that include protection against cancer [1]. The bioactivity of garlic is attributed to a collection of sulfur-containing polysulfanes that are released when the clove is damaged in chemical defence against the invasive threat. In whole, undamaged cloves, the enzyme allinase and its substrate alliin are separated into compartments; however, when the clove is damaged, the allinase and its substrate come into contact to produce allicin. Allicin is unstable, and able to readily undergo thiol/disulfide exchange, or to eliminate to form a number of more stable secondary metabolites that constitute aged or heated garlic preparations [2, 3]. Ajoene (*E/Z*- 4,5,9-trithiadodeca-1,6,11-triene 9-oxide) (see Fig. 1a) is one of these stable rearrangement products of allicin.

Ajoene and its related polysulfane family members have been shown to counter the different stages of cancer. In this regard, they inhibit tumour initiation by various chemical carcinogens (reviewed in [4]), and counter tumour growth by inhibiting proliferation and inducing apoptosis in growing cancer cells (reviewed in [5, 6]). Some of the garlic polysulfanes have been shown to inhibit the more advanced stages of cancer by countering the metastatic process [7–10]. Ajoene displays attractive therapeutic properties, being cytotoxic to cultured cancer cells in the low micromolar range [9, 11–13], and showing a level of selectivity for cancer over normal cells [11–13] while being relatively non-toxic *in vivo* [14].

Ajoene has a rare vinyl disulfide functional group that is seldom found in other natural products. Disulfides are known in biological systems to undergo mixed disulfide exchange reactions with biological thiols, and ajoene and its related family members may mimic and interfere with these processes. Indeed, a number of the garlic polysulfanes have been shown to spontaneously react with glutathione to form GSS-allyl [15–17]. *In vitro* experiments have demonstrated that certain proteins are targeted and *S*-thiolated by garlic polysulfanes at a cysteine residue [18–20]. We found that thiolysis exchange is regioselective for ajoene [21], and unsymmetrical disulfides [22], with the reaction appearing to be driven by the stability of the expelled leaving group [22]. In the case of ajoene, the allylic sulfur is more electrophilic, and hence the site of attack of the incoming thiol nucleophile (see Fig. 1a). We previously synthesised a fluorescent dansyl labelled ajoene analogue called dansyl-ajoene (DP) that contains the fluorescent group strategically placed on the allylic sulfur end of the disulfide in order to ensure its transfer to a protein target during thiolysis exchange [23] (Fig. 1b). We found that ajoene accumulates in the endoplasmic reticulum (ER) of cancer cells, where it interferes with protein folding by *S*-thiolating the exposed cysteine residues of newly synthesised proteins. This leads to an accumulation of misfolded protein aggregates, which activates the unfolded protein response and induces ER stress. Guided by a gene microarray in treated WHCO1 oesophageal cancer cells, we found that the central regulator of the cytotoxicity of the ajoene analogue bisPMB is GADD34/CHOP [24], which is a transcription factor that regulates the unfolded protein response [25, 26]. We found that ajoene *S*-thiolates numerous proteins in cancer cells [23] although only a handful of them have to date been identified. The finding that ajoene has many targets in cancer cells may not be surprising given that the garlic polysulfanes are active on the different stages of cancer, and broadly speaking, claim numerous health benefits. Therefore, understanding the underlying mechanisms behind the bioactivity of ajoene may be greatly facilitated by identification of its cellular protein targets. In this study we identify vimentin as a target that undergoes thiolysis exchange with ajoene in cancer cells. We show that the covalent modification of vimentin by ajoene disrupts the vimentin filamentous network that in turn counters the metastatic phenotype of MDA-MB-231 and HeLa cancer cells. The finding that vimentin is targeted and disrupted by ajoene supports the dietary role of ajoene in the protection and control of metastatic cancer.

## Methods

### Synthesis of Ajoene and Dansyl-Ajoene

Ajoene was synthesised according to the method of Block et al [3] by refluxing allicin in aqueous acetone. The *E*- and *Z*-isomers were then separated by silica gel chromatography. Dansyl-ajoene (DP) was synthesised as an *E/Z*-mixture and characterised according to our previously published method [23].

### Cell lines and treatments

The MDA-MB-231 and HeLa cell lines were purchased from ATCC (HTB-26 and CCL-2) and have been authenticated by IDEXX Bioresearch, UK on the 3/1/2018 (for MDA-MB-231) and the 9/7/2016 (for HeLa). The cells were found to be negative for mycoplasma prior to conducting any experiments. The cells were cultured in Dulbecco’s Modified Eagle medium according to our previously published methods [24]. Cells were seeded at the specified density and allowed to attach overnight prior to adding ajoene or dansyl-ajoene.

### Immunoblot analysis

For detection of vimentin protein from MDA-MB-231 and HeLa cell lysates by immunoblot, standard protocols according to our previously published works were applied [24]. After separation of proteins by SDS-PAGE, proteins were transferred to a 0.2 μm nitrocellulose membranes (Bio-Rad) using conventional methods. After blocking with 5% non-fat milk, the membranes were incubated with the following primary antibodies overnight at 4 °C: anti-vimentin (V9) (1:1000, Santa Cruz for V9 and H84 and Sigma-Aldrich for V4630), anti-GAPDH (1:1000, Santa Cruz). Specific proteins were detected using appropriate horseradish peroxidase-conjugated secondary antibodies and the LumiGLO chemiluminescent reagent (KPL, Biocom Biotech). A protein ladder (Thermo Fisher Scientific, Life Technologies) was used to estimate the molecular weight of proteins. Proteins were visualised using the UVP BioSpectrum™500 Imaging System (UVP, LCC Upland, CA, USA), captured by the CCD camera (Canon Inc) and analysed with the VisionWorks LS Acquisition analysis software (UVP, LCC Upland, USA). For detection of recombinant vimentin protein by immunoblot, the same protocol as above was applied with the following deviation. Recombinant vimentin (5 μg, Peprotech, USA) was made up to 1 μM in PBS, pH 7.4 and treated with DP (100 μM) in 0.1% DMSO for 30 min at room temperature. The untreated sample was treated with 0.1% DMSO alone. Samples were then boiled at 95 °C for 5 min with or without 100 mM DTT (Sigma-Aldrich). The immunoblot was then run as described above and probed with an anti-dansyl primary antibody (1:7500, molecular probes).

### 2D gel electrophoresis

The 2D gel electrophoresis was performed using 7 cm immobilized pH gradient (IPG) strips (Bio-Rad) with a pH range of 3–10. The total protein lysate collected from MDA-MB-231 cells treated with 25 μM DP for 24 h as described above and containing the dansyl-labelled proteins was dissolved under non-reducing conditions in urea lysis buffer (8 M urea, 4% CHAPS, 0.5% Triton X-100, 1x protease inhibitor cocktail tablet (Sigma-Aldrich) and solubilised by gentle agitation on a vortex for 40 min at 20 °C. The protein was quantified using a modified Bradford method [27]. A total of 250 μg protein together with 0.001% bromophenol blue and 1% carrier ampholytes (Bio-Rad) in a volume of 100 μL was loaded on the IPG strips which were rehydrated overnight at 20 °C. The electrofocusing was performed using a Protean IEF Cell (Bio-Rad) with a maximum current of 50 μA per strip and the following settings: 250 V linear voltage for 20 min; 400 V linear voltage for 2 h and a final step of 20,000 (Vh) with a maximum current of 50 μA per strip. The IPG strips were then equilibrated, under non-reducing conditions, in equilibration buffer (6 M urea, 0.375 M Tris HCl (pH 8.8), 2% SDS, 20% glycerol and 0.00 1% bromophenol blue) for 10 min, followed by a brief wash in distilled water, and equilibrated again before separation on a native SDS-PAGE gel. Two protein loaded IPG strips were separated in duplicate. One of them was then subjected to the 2D-immunoblot analyses with the anti-dansyl antibody, as described above, while the other was used for excision of the corresponding protein spots identified using the 2D-immunoblot for MALDI-TOF MS/MS analyses.

### Proteolytic analysis: 2D gel electrophoresis

Gel pieces prepared above were destained with 200 mM NH_4_HCO_3_:acetonitrile 50:50 (Sigma- Aldrich) until clear. Samples were dehydrated and desiccated before reduction with 2 mM triscarboxyethyl phosphine (TCEP; Fluka) in 25 mM NH_4_HCO_3_ for 15 min at room temperature with agitation. Excess TCEP was removed and the samples again dehydrated. Cysteine residues were carbamidomethylated with 20 mM iodoacetamide (Sigma-Aldrich) in 25 mM NH_4_HCO_3_ for 30 min at room temperature in the dark. After carbamidomethylation the samples were dehydrated and washed with 25 mM NH_4_HCO_3_ followed by another dehydration step. Proteins were digested by rehydrating the samples in trypsin (Promega) at 20 ng/μL and incubating at 37 °C overnight. Peptides were extracted from the gel pieces once with 50 μL 0.1% trifluoroacetic acid (TFA) (Sigma-Aldrich). The samples were dried and dissolved in 0.1% TFA, and then purified and concentrated using a C18 ZipTip according to the manufacturers instruction. The purified samples were eluted with 5 mg/mL α-cyano-4-hydroxycinamic acid in 50% ACN:H_2_O containing 0.1% TFA and spotted manually onto a MALDI target plate. MALDI-TOF MS/MS was performed using a 4800 MALDI TOF/TOF system (AB SCIEX) with instrument control through 4000 Series Explorer. Parent spectra were acquired in reflector positive mode at a laser intensity of 4000 arbitrary units using 600 laser shots per spectrum. The scan range of m/z = 800–4000 was used with a grid voltage of 16 kV. Spectra were internally calibrated using trypsin autolytic fragments. Fragmentation data was acquired in positive mode with a deceleration voltage of 1 kV. The spectra were acquired with a laser intensity of 4500 arbitrary units and 1600 shots per spectrum. Database interrogation was performed with the Mascot algorithm using the MSDB database on a GPS workstation.

### Proteolytic analysis: Recombinant vimentin

Purified recombinant vimentin protein, treated with ZA or DP, was prepared as described in 2.3 above. These proteins were run on SDS-PAGE, stained with coomassie and the identified bands were excised from the gel. Gel pieces were treated with Trypsin (Promega) at a final trypsin:protein ratio of 1:20 made up to 50 μL with 50 mM NH_4_HCO_3_ (Sigma-Aldrich). Samples were digested for 18 h at 37 °C. Peptides were then dried by vacuum centrifugation and resuspended in 0.1% formic acid (Sigma-Aldrich) and 2.5% acetonitrile (Anatech) to a final concentration of 500 ng/μL. Samples were then stored at − 80 °C until analysis. Nano-RP LC chromatography was performed using a Dionex Ultimate 3000 nano-HPLC system. LC-MS/MS analysis was conducted with a Q-Exactive quadrupole-Orbitrap mass spectrometer (Thermo Fisher Scientific) coupled with a Dionex Ultimate 3000 nano-HPLC system. The mobile phases consisted of solvent A (0.1% formic acid in water) and solvent B (100% CH_3_CN, 0.1% formic acid). The HPLC fractionated peptides were dissolved in sample loading buffer (2.5% CH_3_CN, 0.1% formic acid) and loaded on a C18 trap column (100 μm × 20 mm × 5 μm). Chromatographic separation was performed with a C18 column (75 μm × 250 mm × 3.6 μm). The mass spectrometer was operated in positive ion mode with a capillary temperature of 250 °C and an applied electrospray voltage of 1.95 kV. Database interrogation was performed by CPGR with the Mascot algorithm using the MSDB database on a GPS workstation.

### Cellular viability assay

Cytotoxicity of ZA was evaluated using the standard MTT cellular viability assay according to our previously published methods [23, 24].

### Immunofluorescence

Cells were immunostained according to our previously published methods [23]. Briefly, MDA-MB-231 or HeLa cells were seeded on sterile coverslips in 6-well culture dishes (5 × 10^5^ cells per well) and allowed to settle overnight. Thereafter, the cells were treated with 20 μM ZA in 0.1% DMSO or DMSO alone (control) for 6 h. Cells were then washed with cold PBS (thrice), permeabilised with methanol at − 20 °C for 5 min and fixed in 4% paraformaldehyde (Sigma-Aldrich) for 5 min at room temperature. Cell sections were then washed with PBS (thrice), incubated in blocking solution (1% BSA in PBS) for 1 h at room temperature, and then incubated with the primary antibodies (anti-vimentin: V9, H84 or V4630) diluted in blocking solution (1:100) overnight at 4 °C in the dark. Sections were then washed with PBS and incubated with the relevant Cy3-labelled secondary antibodies (Jacksons ImmunoResearch, supplied by Amersham, South Africa) diluted in blocking solution (1:500) for 90 min at room temperature in the dark. Sections were then washed with PBS (thrice), mounted using Mowiol 4-88 (Sigma-Aldrich) and stored in the dark at 4 °C until viewing by confocal scanning laser microscopy (Zeiss LSM510NLO).

### Vimentin siRNA transfection

MDA-MB-231 or HeLa cells were seeded in 6-well culture dishes (1 × 10^5^ cells per well) and cultured as described in the general protocol above. The following day, the cells were transfected with 50 nM (MDA-MB-231) or 100 nM (HeLa) vimentin siRNA (Silencer® Select, Life Technologies), using Transfectin Lipid reagent (Bio-Rad) according to the manufacturer’s instruction. After 6 h for MDA-MB-231, and 72 h for HeLa, medium containing the transfection mixture was replaced by fresh culture medium containing 10% FBS and the cells were incubated for 24 h at which time the cells were then used in the wound healing and invasion assays described in the next section. Lysates from these cells were prepared and applied to immunoblotting as described above.

### Vimentin overexpression

MDA-MB-231 cells (5 × 10^5^) or HeLa cells (1 × 10^5^) were seeded in 6-well culture dishes and allowed to attach overnight. The following day, cells were transiently transfected with 1 μg of human vimentin cDNA cloned into pCMV3 (Sino Biological Inc.) using TransFectin lipid reagent (Bio-Rad) according to the manufacturer’s instructions. 4 h later the media containing the transfection mixture was replaced with fresh media containing 10 μM *Z*-ajoene in 0.1% DMSO or 0.1% DMSO alone which was incubated with the cells for a further 24 h.

### Wound healing migration assay

MDA-MB-231 cells (5 × 10^5^) or HeLa cells (1 × 10^5^) were seeded in 6-well culture dishes and allowed to attach overnight. Cells transfected with vimentin siRNA or with pCMV3-vimentin were simultaneously prepared and cultured as described in 2.9 or 2.10 above. After the treatment, several lines were drawn underneath the dishes with a marker as a reference line for wound measurement. Three parallel scratch wounds were then made using a yellow plastic tip. The cells were washed twice with PBS to remove debris, thereafter fresh media was added. Untransfected and transfected cells were then treated with either 0.1% *v*/v DMSO or 10 μM ZA in 0.1% v/v DMSO for 24 h. Images of the cells were taken at the place of the wound at 0 h and 24 h. The wounds were observed by phase contrast using an Olympus CKX41 inverted microscope analysed with AnalySIS getIT software (Olympus, Tokyo, Japan).

### Transwell invasion assay

MDA-MB-231 cells or HeLa cells were transfected with siRNA as described in 2.9 above and cultured overnight in preparation for the transwell invasion assay. Matrigel (BD Biosciences) was thawed overnight at 4 °C. Transwell 6-well plates with permeable cell culture inserts of 12 mm diameter with 8 μm pores (Corning) were also chilled to 4 °C. Matrigel was diluted to 2 mg/mL using serum-free DMEM. Then matrigel (100 μL) was added to the upper compartment of the insert, and the plates were incubated at 37 °C for 2 h to solidify the matrigel. Untransfected and transfected MDA-MB-231 and HeLa cells were trypsinised and resuspended in DMEM. Media was added to both the bottom well (DMEM containing 10% FBS as attractant) and the upper compartment of the insert (DMEM containing 1% FBS). MDA-MB-231 cells (1 × 10^5^) and HeLa cells (1 × 10^5^) were then added to the upper compartment and allowed to attach for 5 h before treatment with ZA (10 μM) in 0.1% v/v DMSO or 0.1% v/v DMSO alone for 24 h. The cells and the matrigel in the upper compartment were then removed gently by wiping with a cotton swab. Those cells on the lower side of the insert membrane were fixed with 5% glutaraldehyde for 10 min at room temperature. The cells were then stained with 1% crystal violet in 2% ethanol at room temperature for 20 min. Thereafter the inserts were immersed thrice in water and dried. The number of cells that invaded through the matrigel were counted in four different fields by visual observation using an Olympus CKX41 inverted microscope analysed with AnalySIS getIT software (Olympus, Tokyo, Japan).

### Computational modelling of vimentin

The structure of the vimentin tetramer PDBID 3KLT was chosen, prepared and modelled using Schrödinger (Release 2017–2 with Maestro, Protein Preparation Wizard, Epik and Jaguar) [28–30]. Empirical pK_a_ calculations, and rigid coordinate QM scans were carried out for cysteine and for cysteine within a short sequence of each chain of vimentin. Empirical pK_a_ calculations were conducted using Epik with default settings at pH 7. There is a limit on the number of atoms (500) that can be used in these calculations, the largest system used was the sequence RQVQSLTCEVDALK (chains A and B included). Calculations were carried out for cysteine, cysteine in the sequence RQVQSLTCEVDALK and cysteine in the sequence TCE. Scans over the C-C-C-S and C-C-S-H dihedral angles were carried out to map the potential energy landscape for each of the cysteine’s in vimentin. These scans were completed using Jaguar with density functional theory (DFT) and the basis B3LYP/6-31G**.

### Statistical analysis

The data was analysed using 1-way ANOVA, multiple comparisons to ascertain the statistical significant differences between untreated and treated samples. Graphpad prism software version 6 was used to assess the significance. *P* < 0.05 samples were considered significant where * *P*-value < 0.05; ** *P*-value < 0.01; *** *P*-value < 0.001.

## Results

### Ajoene targets vimentin in MDA-MB-231 cells

Previous structure-activity studies in our laboratory have identified the vinyl disulfide functional group to be the ajoene pharmacophore that is responsible for cancer cell cytotoxicity [21]. Additionally, we found that the vinyl group plays an important role in enhancing this activity through resonance-stabilisation of the enethiolate leaving group [21, 22]. We further found that the allyl side groups in ajoene are not critical for its cytotoxicity and can be substituted without affecting activity and in some cases improving its activity. Based on these insights, we synthesised a dansyl-labelled ajoene probe called DP [23]. The dansyl label was placed onto the allyl sulfur end to ensure transfer to the protein targets during thiolysis exchange (see Fig. 1B for the scheme). DP was found to be cytotoxic to MDA-MB-231 breast cancer cells with a cytotoxicity IC_50_ of 21 ± 6.2 μM [23], which is in the same range as the parent *Z*-ajoene (ZA) of 14 ± 2 μM [12]. We therefore treated MDA-MB-231 breast cancer cells with 25 μM DP or ZA for 24 h and the lysate was collected, separated by SDS-PAGE and transferred to a nitrocellulose membrane under non-reducing conditions to avoid any cleavage of the disulfide bond and the dansyl label from its target during processing. Any proteins covalently linked to a dansyl group were visualised using an anti-dansyl antibody. Many proteins were found to be dansylated in MDA-MB-231 breast cancer cells while no proteins were detectable in the ZA-treated control sample as expected (Fig. 1c). We had previously established by competition assay that ZA and DP share the same targets [23], and since very few of these targets are known, we attempted to separate and identify them by 2D gel electrophoresis. Although the non-reducing conditions used for this 2D gel were not optimal for separation as the proteins remain partly folded with their disulfide bonds intact, the separation nevertheless identified a few spots, especially below pH 6 and in the 50 to 70 kDa range (Fig. 1d). A prominent band (circled) was excised from the gel and identified by MALDI-TOF MS/MS to be the 57 kDa protein vimentin.

### Validation of the vimentin target

**Fig. 2:**
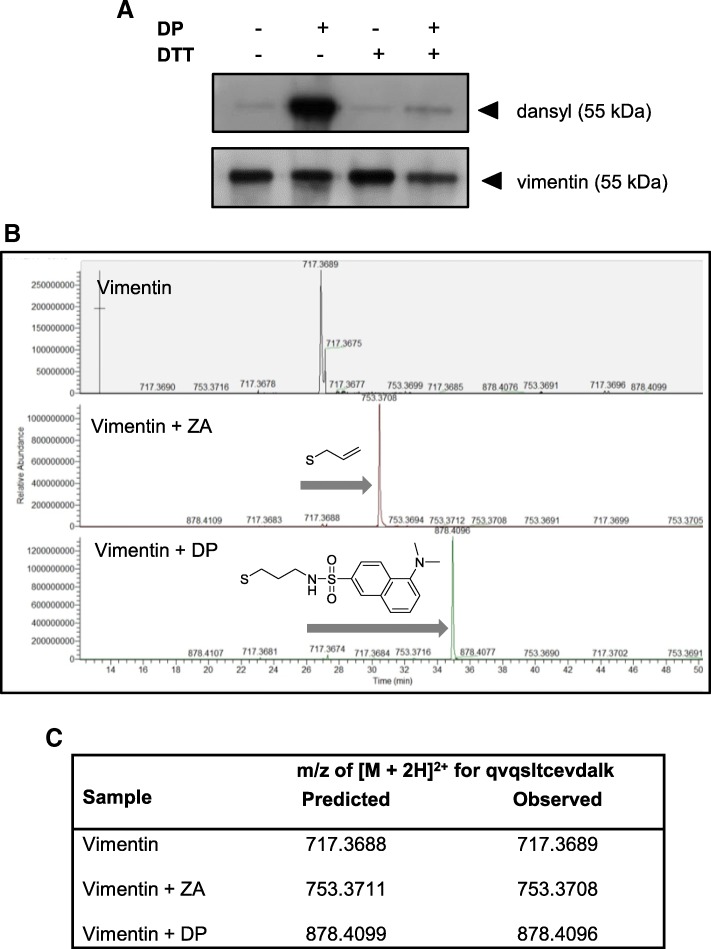
Validation of Vimentin as an Ajoene Target. (**a**) Immunoblot of human recombinant vimentin treated with DP (100 μM) in the absence or presence of DTT (100 mM), probed with a primary anti-dansyl and anti-vimentin (H-84) antibody. (**b**) Human recombinant vimentin was treated with 100 μM ZA or DP and purified by SDS-PAGE. The band excised from the gel was digested with trypsin and fragments were identified by MS/MS MALDI-TOFF mass spectrometry. The Cys-328 containing fragment qvqsltcevdalk was detected in the control and treated samples carrying a 2+ charge where m/z = [M + 2H]2+. (**c**) In the samples treated with ZA or DP, the predicted m/z ratio of the modified fragment was observed

In order to validate that the vimentin protein isolated from MDA-MB-231 cell lysate is a true target of ajoene, we performed a series of experiments on the pure recombinant protein. Recombinant vimentin was incubated with 100 μM DP or ZA in PBS buffer for 1 h. The protein was then migrated on SDS-PAGE and transferred to a membrane for visualisation of any dansyl-incorporation using the anti-dansyl antibody (in the case of DP treatment, see Fig. 2a). The dansyl label from DP was clearly seen to be covalently attached to the vimentin protein under non-reducing conditions (−DTT). In agreement with the attachment of the dansyl label through a disulfide bond, it was cleaved following treatment of the protein with the reducing agent dithiothreitol (+DTT). To identify the specific ajoene binding site amino acid on the vimentin protein, high resolution mass spectrometry was performed. Recombinant vimentin treated with ZA or DP, was excised from the gel and digested with trypsin to produce vimentin peptide fragments. The peptides were then separated chromatographically using a C18 column and identified by MALDI-TOF MS/MS mass spectrometry. Database interrogation was performed to identify the peptide fragment containing Cys-328 that was found carrying a 2+ charge (Fig. 2b). In the treated samples, this Cys-328 containing fragment was identified carrying the expected mass from ZA or DP (see Fig. 2c and Additional file 1: Figure S1). Taken together, these results support the finding that vimentin is a target of ajoene in MDA-MB-231 cells and that ajoene covalently binds to vimentin at Cys-328 by *S*-thiolation.

### Ajoene disrupts the vimentin filament network in cells

**Fig. 3:**
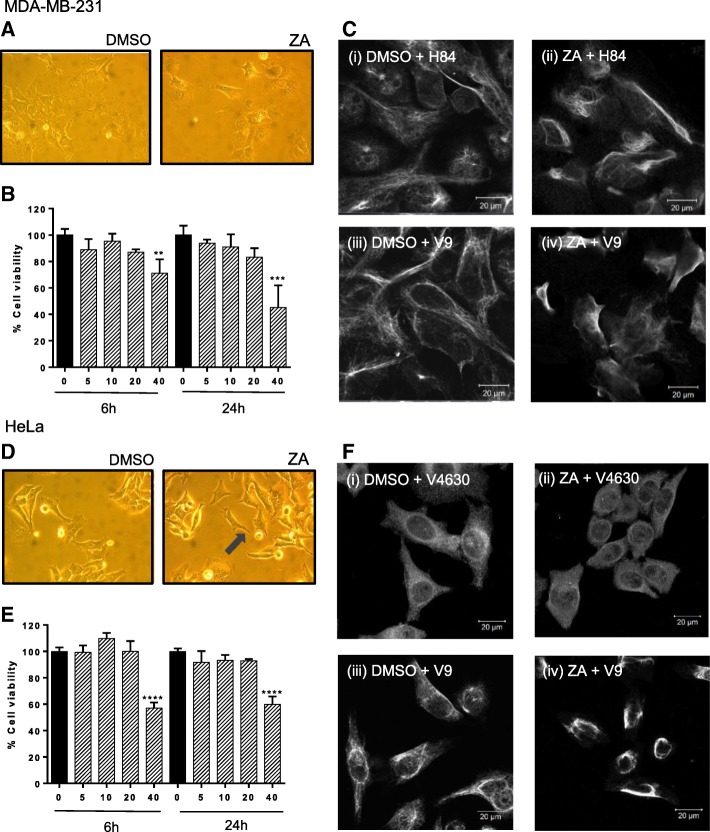
Ajoene Disrupts the Vimentin Filament Network in MDA-MB-231 and HeLa cells. 40x Phase contrast images of MDA-MB-231 (**a**) or HeLa (**d**) cells treated with DMSO (control) or 10 μM ZA in DMSO for 24 h. Cell viability assay: MDA-MB-231 (**b**) or HeLa (**e**) cells treated with DMSO (control) or with ZA (0, 5, 10, 20 or 40 μM) for 6 h or 24 h. Immunofluorescence: MDA-MB-231 (**c**) or HeLa (**f**) cells treated with 20 μM ZA for 6 h, then fixed and immunostained with vimentin primary antibodies (V9, H84 or V4630). Control cells treated with DMSO alone. Images obtained by confocal scanning laser microscopy

The *S*-thiolation of vimentin by ajoene may be seen as a post-translational modification that leads to a change in the overall protein structure. Vimentin is a structural protein and a member of the intermediate filament family of proteins that is ubiquitously expressed in normal mesenchymal cells [31]. Vimentin monomers self-assemble into filaments that form scaffolds and organise the cytoplasmic space to define and maintain the cellular architecture [32]. Vimentin is considered a cancer marker as it is overexpressed in most epithelial cancers undergoing epithelial to mesenchymal transition (EMT), and its overexpression correlates well with accelerated tumour growth, invasion, angiogenesis and poor prognosis [33–35]. We investigated whether the covalent attachment of ZA to vimentin may affect the filamentous network. Non-cytotoxic treatment conditions of ZA were first selected as it was not the intention to induce apoptosis in cells, which would lead to protein degradation and an impairment of vimentin function by virtue of apoptosis. We first assessed cell viability of two cancer cell lines, MDA-MB-231 and HeLa cells, using the MTT assay and selected two treatment conditions for further investigations: 20 μM ZA for 6 h (treatment condition 1); or 10 μM ZA for 24 h (treatment condition 2) (Fig. 3b and e). Although the cells under these treatment conditions were considered viable by the MTT assay, the cellular morphology for the HeLa cells, but not for the MDA-MB-231 cells, appeared altered (shown for treatment condition 2, Fig. 3a and d). The treated cells appeared slightly shrunken and sharpened at the edges (Fig. 3d, see arrow), although the proliferation rates were unchanged. Treated cells were then fixed and immunostained with different vimentin primary antibodies to visualise the cellular vimentin network (Fig. 3c and f). In the control cells, defined networks of filamentous vimentin fibres were observed with the different antibodies for vimentin. V9 recognises full length vimentin and these cells showed distinct filaments. H84 recognises an epitope corresponding to amino acids 1-84 mapping at the N-terminus of vimentin which also appears filamentous but more diffuse. V4630 staining did not appear filamentous but more granular and dispersed uniformly throughout the cytoplasm. Using either of the antibodies, the cells treated with ZA showed condensed filaments that did not extend far into the intracellular space. The effect appeared most pronounced when viewed with the antibody for intracellular vimentin (V9) which is the antibody most commonly used to immunostain vimentin, although in all cases, the filaments appeared shrunken and condensed. An appropriate conclusion is that ZA may be inhibiting the proper formation of the vimentin filament network in a way that is important in organising the cytoplasmic space, this could explain the somewhat shrunken morphology of treated HeLa cells.

### Ajoene induces increased vimentin expression

**Fig. 4:**
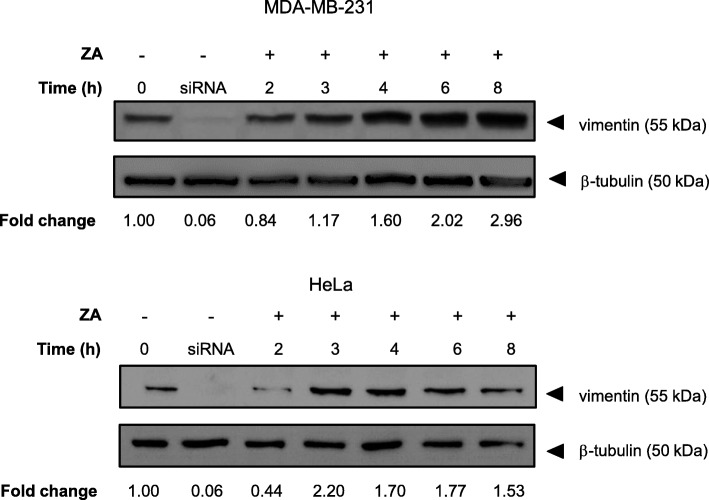
Ajoene Induces Increased Expression of Vimentin. MDA-MB-231 (top) or HeLa (bottom) cells were treated with either DMSO (control) or 10 μM ZA in DMSO up to 8 h. Proteins collected from the cell lysate were separated by SDS-PAGE and vimentin expression was quantified by immunoblot probed with a primary anti-vimentin antibody (V9). The blots shown are a representative experiment of two independent determinations

We investigated whether ajoene may have an effect on the expression levels of vimentin protein by treating MDA-MB-231 or HeLa cells with ZA under non-cytotoxic conditions. At various time-points up to 8 h, cell lysate was collected and total vimentin protein expression was quantified by immunoblot. Surprisingly, and apparently contradictory to the role that vimentin plays in metastasis, a time-dependent increase in total vimentin protein was observed (Fig. 4).

### Ajoene inhibits invasion and migration

**Fig. 5:**
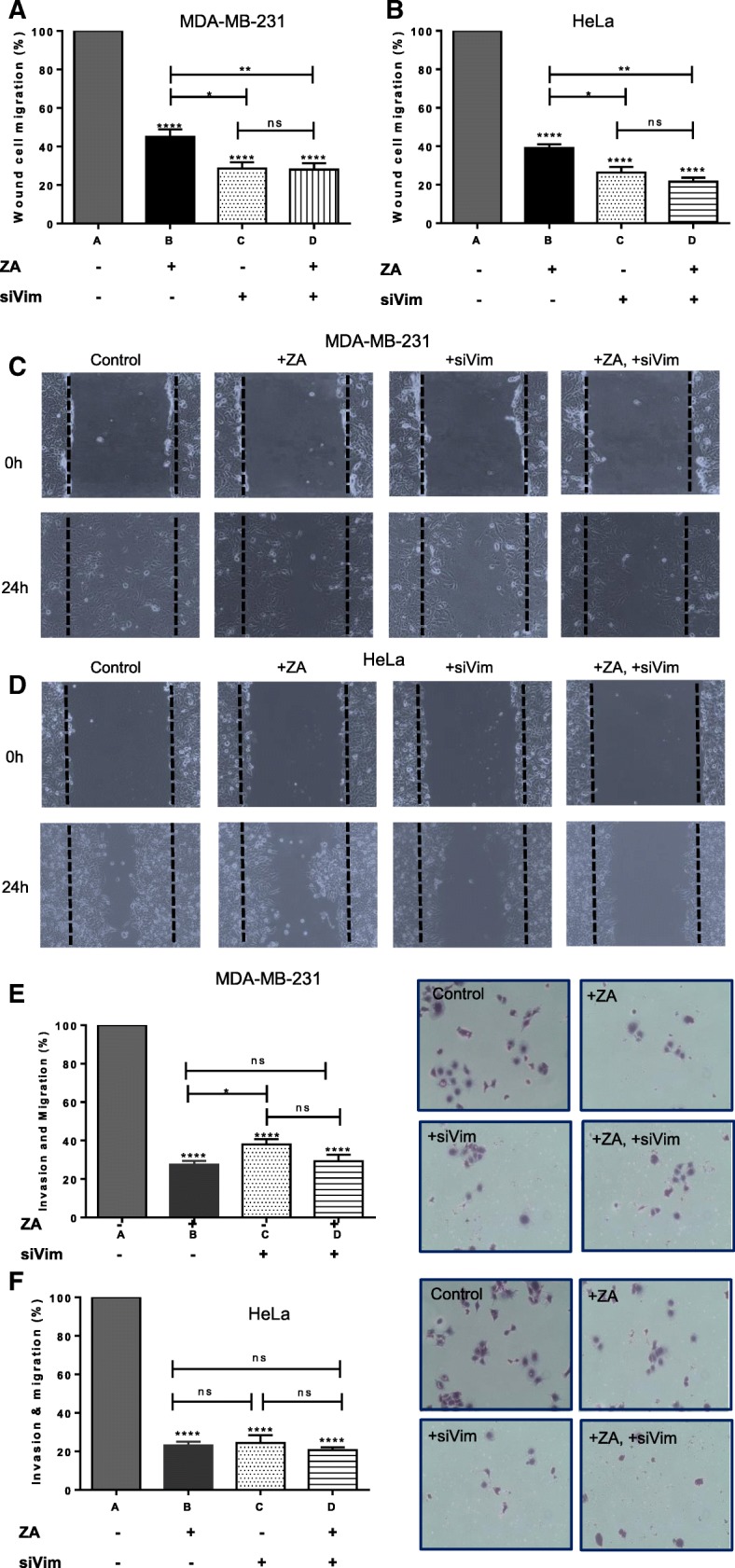
Ajoene Inhibits the Invasion and Migration of MDA-MB-231 and HeLa Cells. Wound healing assay: Following introduction of a scratch wound into the cell layer of (**a** and **c**) MDA-MB-231 or (**b** and **d**) HeLa cells; native cells, or cells silenced for vimentin expression were incubated with 10 μM ZA in DMSO or DMSO alone for 24 h. Migration into the wound was then quantified using Image J software. Transwell invasion and migration assay: (**e**) MDA-MB-231 cells or (**f**) HeLa cells were transfected with vimentin siRNA and treated as described above for 24 h. The ability of cells to invade and migrate through the matrigel membrane was quantified by counting the crystal violet stained cells, data displayed as mean ± SD. The results of a single representative experiment is shown; however experiments were performed in duplicate

Tumour cells acquire the ability to infiltrate blood or lymphatic vessels via EMT, which is widely believed to increase tumour aggressiveness and aid in metastasis. These events are facilitated by the reduction in the cell-cell adhesion molecule E-cadherin; and upregulation of the more plastic mesenchymal proteins such as vimentin, N-cadherin, matrix metalloproteinase (MMP)-2, and smooth muscle actin [36]. Vimentin is overexpressed in many cancers and its overexpression is frequently associated with an increased migratory and invasive capacity [33]. Moreover, some of the garlic organosulfur compounds are reported to inhibit the invasion and migration of cancer cells, although this has not been shown for ajoene. Thus, we decided to investigate whether ajoene may exert anti-metastatic activity in cancer cells by looking at the MDA-MB-231 and HeLa cell lines. To this end, MDA-MB-231 or Hela cells were treated with a non-cytotoxic concentration of ZA, and anti-metastatic activity was assessed by the wound healing migration assay (Fig. 5a-d) as well as by a transwell invasion assay (Fig. 5e and f). The migration experiment involves introducing a scratch wound into the cell monolayer and quantifying the ability of the cells to migrate into this wound in the presence of ZA. In the invasion assay, the ability of the cancer cells to degrade and move through the transwell membrane matrix is assessed. As a control, we included in these experiments cells in which vimentin expression had been transiently silenced with vimentin siRNA. Importantly, it was found that cells lacking the vimentin protein were unable to migrate into the scratch wound, findings that are consistent with the role of vimentin in EMT and that confirm a previous literature report [37] (Fig. 5a-d). These cells also showed much decreased ability to break down and invade the membrane matrix (Fig. 5e and f). The presence of ZA was found to inhibit the migration of cells into the scratch wound, and to inhibit invasion of the cells through the membrane. Interestingly, when ZA was added to cells that lacked vimentin protein (siVim cells), no further reduction in invasion or migration was observed (compare columns C and D). This suggests the cell target of ajoene inhibition of cell motility and invasion is vimentin. In comparing the effects of adding ZA alone, to vimentin deficient cells (compare columns B and D) a small significant decrease was seen in the wound healing assay but not in the invasion assay. This additive effect may be explained by the observation that ZA compromises the vimentin network, although it does not completely dismantle it (as observed in immunofluorescence experiments in Fig. 3), while depleting vimentin has a more pronounced effect and is statistically significant in the wound healing experiment. Silencing vimentin blocks more than ZA in the wound healing experiment but in invasion ZA is more potent, possibly because it may also inhibit other processes associated with EMT which is supported in the literature for other garlic organosulfur compounds.

### Ajoene partially rescues the phenotype of vimentin overexpression

**Fig. 6:**
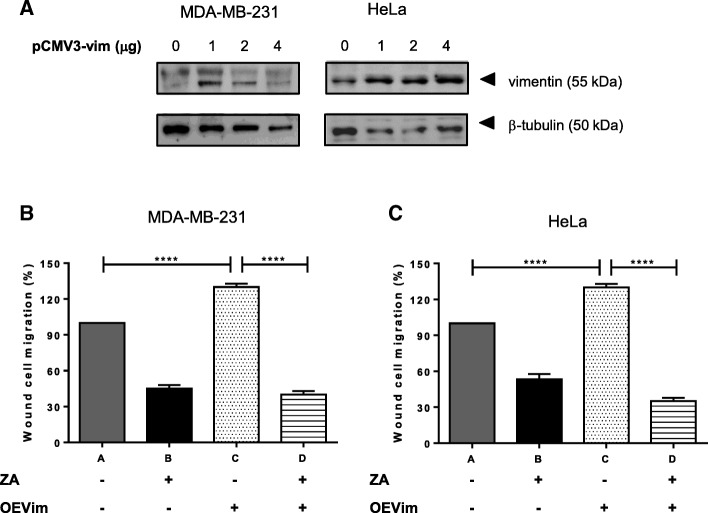
Ajoene Rescues the Enhanced Migratory Potential of Vimentin Overexpressing Cells. (**a**) Vimentin was transiently overexpressed using human vimentin cDNA cloned into pCMV3, in both HeLa and MDA-MB-231 cells as shown and quantified by immunoblot. A scratch wound was then introduced into MDA-MB-231 (**b** and **d**) or HeLa (**c** or **e**) cells and 10 μM ZA in DMSO or DMSO alone was incubated with the cells for 24 h. Migration into the wound was then quantified using Image J software. The results of a single representative experiment is shown; however experiments were performed in duplicate

To further investigate whether vimentin is a functionally relevant target of ajoene’s anti-metastatic activity, we transiently overexpressed vimentin in HeLa and MDA-MB-231 cells (Fig. 6a). A scratch wound was then introduced into the cell monolayer, followed by addition of 10 μM *Z*-ajoene in DMSO or DMSO alone for 24 h. In agreement with the literature [33], vimentin overexpression was found to increase the migratory potential of both cancer cell lines up to 130%. Interestingly, ajoene completely countered the increased migratory potential in both cell lines, caused by vimentin overexpression (Fig. 6). This experiment strongly suggests that the anti-migratory activity of ajoene is mediated through the vimentin target.

### Computational modelling of the vimentin tetramer

**Fig. 7:**
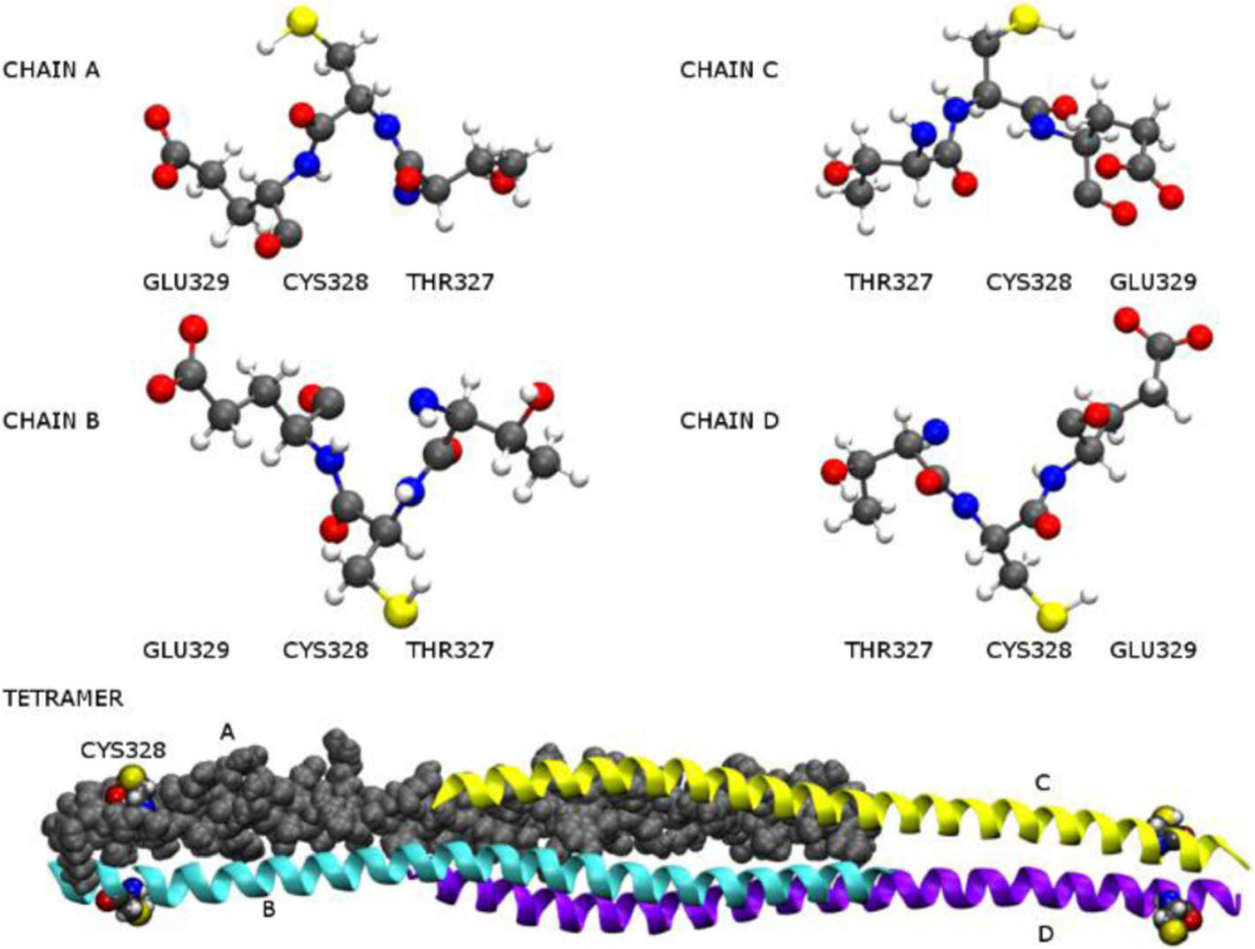
Computational modelling of the Vimentin tetramer showing the environment of Cys-328. The structure of the vimentin tetramer (PDBID 3KLT) was chosen, prepared and modelled using Schrödinger software. The structure of the tetramer is labelled and shown using a space filling representation for chain (**a**) (grey) and modified cartoon representation for chains (**b**, **c**, **d**) (cyan, yellow and magenta), The four cysteine thiols are coloured yellow and are exposed at the termini of the tetramer. The environment for the cysteine of each chain in the tetramer is illustrated. The thiol hydrogen points in the direction of the glutamate in chain (**a**), towards the carbonyl oxygen in chains (**c**, **d**) and towards Gln-324 in chain (**b**)

It is proposed that vimentin monomers assemble into parallel dimers that in turn assemble antiparallel and staggered into tetramers which are considered the structural units for vimentin polymerisation [36–38]. The vimentin tetramer (Protein data base 3KLT) which is composed of four vimentin chains was chosen, prepared and computationally modelled using Schrödinger modelling software (Fig. 7). Alternative PDB structures were considered to be incomplete by either missing the cysteine or present as vimentin dimers only. For a cysteine residue to be reactive we expect it to be accessible. From a visual inspection, there is no concave binding domain for substrates in the vicinity of Cys-328; however all four of the cysteine residues, found at the two termini of the tetramer, appear exposed and pointing outwards. A cysteine thiolate is a superior nucleophile to a thiol and the ease of deprotonation is reflected in its p*K*_a_. Empirical p*K*_a,_ calculations, and rigid coordinate quantum mechanical scans were carried out for cysteine and cysteine from the selected sequence TCE. Empirical p*K*_a,_ was also calculated for cysteine in the sequence RQVQSLTCEVDALK. Scans over the cysteine CCCS and CCSH dihedral angles were carried out to map the potential energy landscape for each cysteine in vimentin. The p*K*_a_ of cysteine is 8.5 and a reduction in the p*K*_a_ of a cysteine residue requires assistance by amino acids in the surrounding environment [38–40]. Catalytic cysteines, for example in peroxiredoxins and protein tyrosine phosphatases, have very low p*K*_a_ in the ranges 4.6 - 5.5 and 4.5 - 5.9, respectively [41, 42]. Nearby negatively charged amino acids that may assist in this regard by general base catalysis could include Glu-329 and Asp-331, and these were included in the empirical calculations (as per sequence indicated in Additional file 2: Figure S2). The empirical p*K*_a_ calculations were found to be very similar to the experimental p*K*_a_ for cysteine in all models (Additional file 2: Table S1). A 14 amino acid sequence, RQVQSLTCEVDALK of chains A and B containing both Glu-329 and Asp-331, had a slightly lower calculated p*K*_a_ but no significance can be assigned to this (8.47 ± 2.22). Although the cysteine CCCS and CCSH torsion angles within the tetramer crystal structure are very different (Additional file 2: Table S1) further investigations of the energies over dihedral conformations of each cysteine in chains A-D showed that the low energy and high energy regions are similar in all chains with the expected CCCS staggered low energy conformations available (Additional file 2: Figure S2). We therefore conclude that all cysteines are equally reactive in the vimentin tetramer and that there is no obvious preference for general base assisted catalysis to favour thiolate formation. It would appear therefore that the apparent reactivity of Cys-328 towards ajoene is dependent solely on its accessibility.

## Discussion

Cancer is a hyper-proliferative disease that results in over six million deaths per year. Most malignancies are diagnosed and treated at an advanced stage with poor prognosis making cancer prevention an attractive intervention strategy. Several lines of evidence suggest that many cancers are preventable, as their causation is largely exogenous, with diet and lifestyle playing an important role. In this regard, garlic is a medicinal plant that has been used for centuries for its beneficial health effects that include protection against cancer. Its anticancer activity may be ascribed to the organosulfur compounds that are found in crushed garlic preparations, of which ajoene is a family member. These compounds have been shown to inhibit different stages of cancer, supporting our findings that ajoene has multiple protein targets in cancer cells [23]. The majority of these targets are ER-associated, as strong co-localisation was observed between our fluorescently-labelled ajoene analogue, DP, and dyes specific for the ER [23].

The thiol/disulfide exchange reaction between a protein sulfhydryl group and glutathione is well documented in biological systems [43, 44]. This process, called glutathionylation, is reversible and occurs spontaneously under oxidative stress conditions, or can be enzyme catalysed [43]. The capping of protein cysteine residues with glutathione is thought to protect proteins against irreversible oxidative damage, as well as being a post-translational modification important in the regulation of cellular functions [43–47]. The GSSG:GSH ratio is an important indicator of the redox status of the cell, and the non-enzymatically driven extent of protein glutathionylation will vary accordingly: a higher ratio will promote glutathionylation while a lower ratio will result in deglutathionylation and release of GSH. It is reported that approximately 50% of the glutathione in the ER is bound to proteins via glutathionylation [48] compared to less than 1% in other locations [49, 50] due to this organelle being more oxidising in nature. Indeed, it was in the ER that ajoene was found to accumulate [23, 24]. The garlic polysulfanes, including ajoene, may *S*-thiolate cysteine residues in redox-sensitive proteins in a manner similar to glutathionylation [51], and this is supported by our findings that ajoene *S*-thiolates numerous proteins in cancer cells, possibly in competition with GSSG. Based on the presence of a vinyl disulfide (as a better leaving group due to resonance stabilisation of the enethiolate released during thiolysis exchange), ajoene would be expected to be a superior thiolating agent in *S*-thiolation compared to GSSG. Therefore, proteins that are susceptible to glutathionylation are probably the targets of ajoene. In support of this, the proteins identified to date to be *S*-thiolated by garlic organosulfur compounds (tubulin, glutathione reductase and sulfur transferases) are all proteins reported to be glutathionylated [43, 47, 52, 53].

Here we report that vimentin is a newly identified target of ajoene. Vimentin is important in maintaining the structural integrity of normal cells and in epithelial-to-mesenchymal transition. In cancer, vimentin is a driver of cancer progression and contributes to the invasive phenotype of metastatic cancer cells [54, 55]. In addition, vimentin has been shown to be a component of the attachment and uptake complex of several viruses, for example HPV [56]. The Cys-328 residue in vimentin has previously been identified as a site prone to oxidative modification by small molecule electrophiles, an example being withaferin A, a steroidal lactone found in the medicinal plant *Withania somnifera,* that reacts via michael addition of its enone with Cys-328 [57], and this modification was found to mediate antiangiogenic effects [58]. In another example, Cys-328 was found to be oxidatively modified by the electrophilic signalling lipid PGA1 which contains the cyclopentenone structural motif [59]. In the current study we have found that the natural dietary compound ajoene targets vimentin in metastatic MDA-MB-231 cells by covalent oxidation at Cys-328. From a visual inspection of the crystallised vimentin tetramer, there does not appear to be any concave binding site for substrates in the vicinity of Cys-328. This correlates with the observation that diverse electrophilic structures that include a peptide, steroid, lipid and a polysulfane are able to successfully access and oxidise Cys-328. As we did not find any apparent preference for general base assisted catalysis in the vicinity of Cys-328, and empirical p*K*_a_ calculations failed to reveal preference for thioate formation of any of the cysteines in the tetramer. A reasonable conclusion therefore is that the reactivity of Cys-328 to diverse oxidising and electrophilic agents is probably due to its accessibility.

Vimentin filaments are important in organising the cellular architecture and are described as being dynamic, motile and plastic [60–62]. These fluid properties provide mechanisms for their reorganisation and assembly in response to the requirements of the cell; being it adhesion, migration or signalling. The first level of organisation is the formation of coiled dimers that are arranged in parallel [63]. They assemble half-staggered and anti-parallel in an A_11_ manner [64] into tetramers, that are considered the basic structural units for further vimentin polymerisation [61, 64, 65]. Any exchange between tetrameric units is dynamic and occurs end-to-end at any point along the filament length [33, 66]. With cysteine cross-linking agents, it has been possible to link together staggered vimentin dimers [67], and to crosslink vimentin to other filament proteins [32, 68]. Pérez-Sala *et al* found that crosslinking vimentin stabilises the intracellular network and protects it from disruption by electrophilic and oxidising agents [69] thereby showing how reduced Cys-328 is important in the overall stabilisation of the network. In the absence of crosslinking agents, the inter-cysteine distance between tetramers is proposed to be too long to support disulfide bond formation and elemental zinc may bridge the two cysteine residues to stabilise this network *in vivo* [69]. We show that ajoene oxidises Cys-328 of vimentin in MDA-MB-231 and HeLa cells which disrupts the filamentous network and affects the invasive and migratory potential of these cells. Other members of the garlic polysulfane family namely SAMC [7], DADS [8, 10] and DATS [70] are reported to inhibit invasion and migration in different cancer cell lines; and SAMC [7, 71], SAC [72], DATS [70, 73] and ajoene [9] have all been shown to inhibit metastasis *in vivo* in mouse models for cancer [9]. While the antimetastatic activity for ajoene has been demonstrated *in vivo*, this is the first report to demonstrate it in cancer cell lines. Garlic organosulfur compounds have been shown to reverse EMT by inactivating the β-catenin pathway by increasing the expression of the epithelial marker E-cadherin, and decreasing the expression of the mesenchymal markers vimentin, N-cadherin and snail [7, 8], as well as downregulating MMP-2/9 [8, 70]. This is the first report that ajoene directly targets and covalently modifies vimentin in cancer cells and it is therefore not known whether vimentin targeting also occurs for other garlic organosulfur compounds; and conversely whether inhibition of other EMT processes may also occur for ajoene.

Vimentin is a cancer marker that is overexpressed in neoplasms undergoing epithelial to mesenchymal transition. Moreover, its overexpression correlates well with the metastatic phenotype. Our finding that ajoene increases the expression of vimentin in cancer cells is therefore surprising and contradictory to the role that vimentin plays in progression of metastatic disease. Indeed, we found that artificial overexpression of vimentin in both cancer cell lines caused enhanced migration up to 130%. In support of ajoene binding to vimentin, and inhibiting its proper function, the enhanced migratory effect observed in vimentin overexpressing cells was completely inhibited by ajoene. Therefore, although ajoene causes a time-dependent increase in vimentin expression, it is importantly inhibiting the vimentin-dependent increase in migration. We argue that ajoenes increased vimentin expression may be a response to restore the malfunctioning vimentin network. However, due to the continued presence of ajoene, this newly synthesised vimentin does not lead to enhanced migration (in fact reduced migration). In other words, the newly synthesised vimentin does not form functional filaments. A similar contradictory effect has been observed before by Dirsch et al [74]. In that report, ajoene was found to inhibit Cox-2 enzyme activity with a simultaneous increase in the Cox-2 protein and mRNA levels. To our knowledge, our vimentin finding is therefore the second example in the literature, where ajoene has been found to target and inhibit a protein, with a simultaneous increase in its expression.

## Conclusions

The ability of ajoene to covalently bind to Cys-328 of vimentin in cancer cells, causes the filaments become condensed and disrupted. This appears to also cause a shrinking of the cellular morphology. Ajoene-treated cancer cells are less able to migrate and invade the membrane matrix than untreated cancer cells. This antimetastatic activity is related to the ability of ajoene to bind to vimentin as removal of the target countered ajoenes antimetastatic activity. Moreover, ajoene rescued the enhanced migratory potential observed upon artificial vimentin overexpression. Taken together, the findings support the role of ajoene as a natural dietary phytochemical able to offer protection against metastatic cancer, mediated through binding to the vimentin target.

## Additional file

Additional file 1: **Figure S1**. Calculation of vimentin mass fragments. The peptide QVQSLTCEVDALK containing Cys-328 was detected carrying a 2+ charge. The predicted mass of the same fragment modified by DP or ZA was then calculated accordingly. (PPTX 40 kb)

Additional file 2: **Figure S2**. Computational modelling of the torsional conformational energies of the cysteine motifs extracted from the vimentin tetramer. The structure of the vimentin tetramer (PDBID 3KLT) was chosen, prepared and modelled using Schrödinger. A short sequence motif (TCE) was chosen for chain A-D and this was modelled using Jaguar’s rigid coordinate scan. The dihedrals C-C-C-S and C-C-S-H define the conformational space of the cysteine. Maps for chain A-D are shown with a duplicated phase space range for improved visualisation. Low energy and high energy regions are similar in all chains. **Table S1.** A summary of p*K*_a_ predictions and torsional angles for model systems of cysteine and cysteine in vimentin. (PPTX 377 kb)

## Abbreviations

ACN: Acetonitrile
BSA: Bovine serum albumen
CHOP: C/EBP homologous protein
DMSO: Dimethyl sulfoxide
DP: Dansyl-ajoene
DST: Density function theory
DTT: Dithiothreitol
DTT: Dithiothreitol
EMT: Epithelial to mesenchymal transition
ER: Endoplasmic reticulum
FBS: Foetal bovine serum
GSH: Glutathione
IPG: Immobilized pH gradient
kDa: Kilodalton
MALDI TOF MS/MS: Matrix-assisted laser desorption time of flight tandem mass spectrometry
MMP-9: Matrix metalloproteinase 9
MW: Molecular weight
PAGE: Polyacrylamide gel electrophoresis
PBS: Phosphate buffered saline
PBS: Phosphate buffered saline
PDB: Protein data base
QM: Quantum mechanical
TFA: Trifluoroacetic acid
TNBC: Triple negative breast cancer
ZA: *Z*-ajoene
