# Supplementary material for: The garlic compound ajoene covalently binds vimentin, disrupts the vimentin network and exerts anti-metastatic activity in cancer cells
Source: BMC Cancer. 2019 Mar 20;19:248. doi: 10.1186/s12885-019-5388-8 (PMC6425727; doi:10.1186/s12885-019-5388-8)
Supplement: Supplementary file 1 — Figure S1. Calculation of vimentin mass fragments. The peptide QVQSLTCEVDALK containing Cys-328 was detected carrying a 2+ charge. The predicted mass of the same fragment modified by DP or ZA was then calculated accordingly. (PPTX 40 kb) [file 12885_2019_5388_MOESM1_ESM.pptx]

## Slide 1
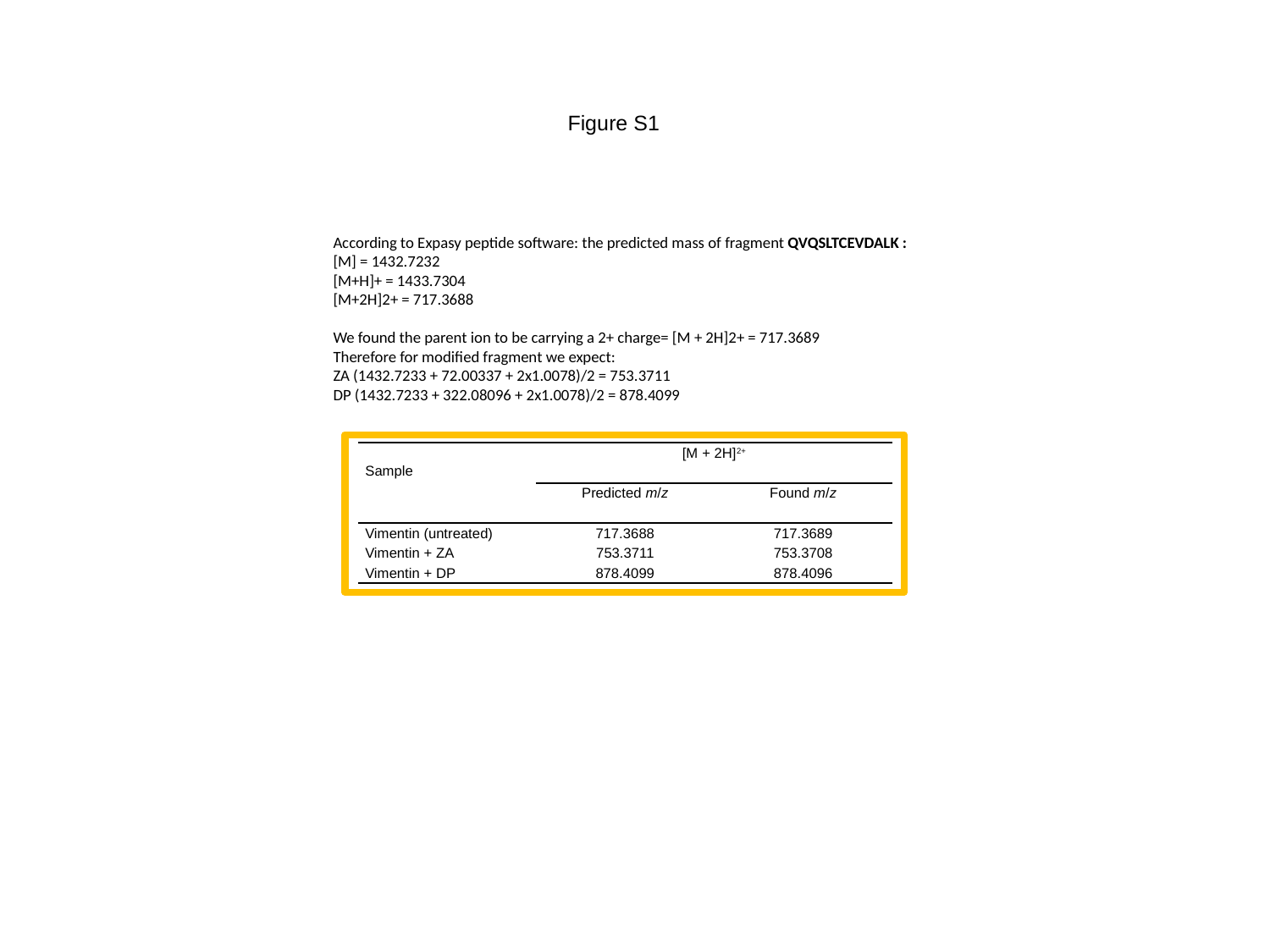

Figure S1
According to Expasy peptide software: the predicted mass of fragment QVQSLTCEVDALK :
[M] = 1432.7232
[M+H]+ = 1433.7304
[M+2H]2+ = 717.3688
We found the parent ion to be carrying a 2+ charge= [M + 2H]2+ = 717.3689
Therefore for modified fragment we expect:
ZA (1432.7233 + 72.00337 + 2x1.0078)/2 = 753.3711
DP (1432.7233 + 322.08096 + 2x1.0078)/2 = 878.4099
| Sample | [M + 2H]2+ | |
| --- | --- | --- |
| | Predicted m/z | Found m/z |
| Vimentin (untreated) | 717.3688 | 717.3689 |
| Vimentin + ZA | 753.3711 | 753.3708 |
| Vimentin + DP | 878.4099 | 878.4096 |
