# Supplementary material for: The garlic compound ajoene covalently binds vimentin, disrupts the vimentin network and exerts anti-metastatic activity in cancer cells
Source: BMC Cancer. 2019 Mar 20;19:248. doi: 10.1186/s12885-019-5388-8 (PMC6425727; doi:10.1186/s12885-019-5388-8)
Supplement: Supplementary file 2 — Figure S2. Computational modelling of the torsional conformational energies of the cysteine motifs extracted from the vimentin tetramer. The structure of the vimentin tetramer (PDBID 3KLT) was chosen, prepared and modelled using Schrödinger. A short sequence motif (TCE) was chosen for chain A-D and this was modelled using Jaguar’s rigid coordinate scan. The dihedrals C-C-C-S and C-C-S-H define the conformational space of the cysteine. Maps for chain A-D are shown with a duplicated phase space range for improved visualisation. Low energy and high energy regions are similar in all chains. Table S1. A summary of pKa predictions and torsional angles for model systems of cysteine and cysteine in vimentin. (PPTX 377 kb) [file 12885_2019_5388_MOESM2_ESM.pptx]

## Slide 1
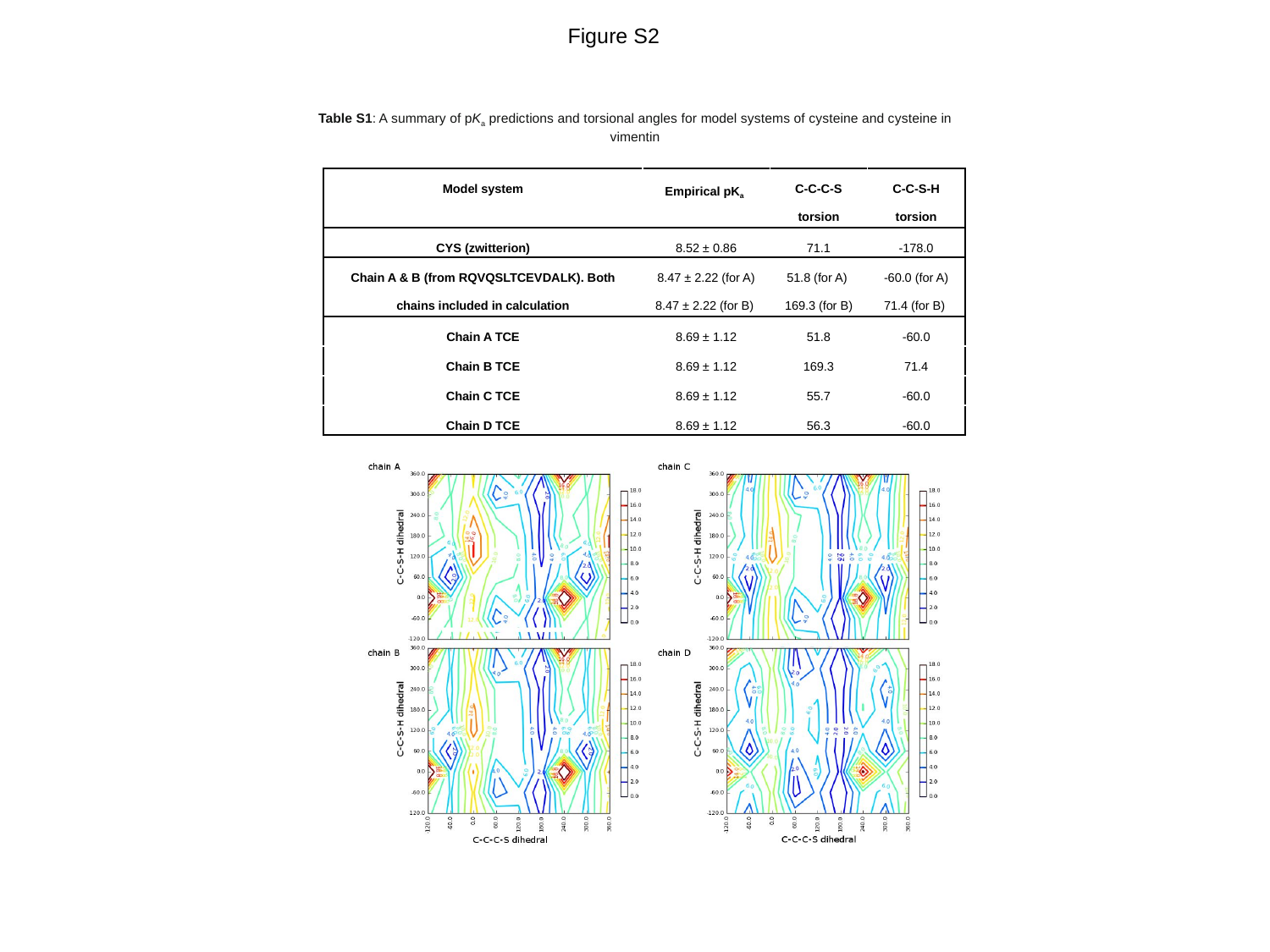

Figure S2
Table S1: A summary of pKa predictions and torsional angles for model systems of cysteine and cysteine in vimentin
| Model system | Empirical pKa | C-C-C-S torsion | C-C-S-H torsion |
| --- | --- | --- | --- |
| CYS (zwitterion) | 8.52 ± 0.86 | 71.1 | -178.0 |
| Chain A & B (from RQVQSLTCEVDALK). Both chains included in calculation | 8.47 ± 2.22 (for A) 8.47 ± 2.22 (for B) | 51.8 (for A)  169.3 (for B) | -60.0 (for A) 71.4 (for B) |
| Chain A TCE | 8.69 ± 1.12 | 51.8 | -60.0 |
| Chain B TCE | 8.69 ± 1.12 | 169.3 | 71.4 |
| Chain C TCE | 8.69 ± 1.12 | 55.7 | -60.0 |
| Chain D TCE | 8.69 ± 1.12 | 56.3 | -60.0 |
